# An Artificial Neural Network Estimation of Gait Balance Control in the Elderly Using Clinical Evaluations

**DOI:** 10.1371/journal.pone.0097595

**Published:** 2014-05-16

**Authors:** Vipul Lugade, Victor Lin, Arthur Farley, Li-Shan Chou

**Affiliations:** 1 Department of Human Physiology, University of Oregon, Eugene, Oregon, United States of America; 2 Rehabilitation Medicine Associates of Eugene-Springfield, P.C., Eugene, Oregon, United States of America; 3 Department of Computer and Information Sciences, University of Oregon, Eugene, Oregon, United States of America; University of Jaén, Spain

## Abstract

The use of motion analysis to assess balance is essential for determining the underlying mechanisms of falls during dynamic activities. Clinicians evaluate patients using clinical examinations of static balance control, gait performance, cognition, and neuromuscular ability. Mapping these data to measures of dynamic balance control, and the subsequent categorization and identification of community dwelling elderly fallers at risk of falls in a quick and inexpensive manner is needed. The purpose of this study was to demonstrate that given clinical measures, an artificial neural network (ANN) could determine dynamic balance control, as defined by the interaction of the center of mass (CoM) with the base of support (BoS), during gait. Fifty-six elderly adults were included in this study. Using a feed-forward neural network with back propagation, combinations of five functional domains, the number of hidden layers and error goals were evaluated to determine the best parameters to assess dynamic balance control. Functional domain input parameters included subject characteristics, clinical examinations, cognitive performance, muscle strength, and clinical balance performance. The use of these functional domains demonstrated the ability to quickly converge to a solution, with the network learning the mapping within 5 epochs, when using up to 30 hidden nodes and an error goal of 0.001. The ability to correctly identify the interaction of the CoM with BoS demonstrated correlation values up to 0.89 (P<.001). On average, using all clinical measures, the ANN was able to estimate the dynamic CoM to BoS distance to within 1 cm and BoS area to within 75 cm^2^. Our results demonstrated that an ANN could be trained to map clinical variables to biomechanical measures of gait balance control. A neural network could provide physicians and patients with a cost effective means to identify dynamic balance issues and possible risk of falls from routinely collected clinical examinations.

## Introduction

Over one third of adults over the age of 65 will fall each year [1]. Falls are not only associated with injury and morbidity, but also reductions in physical, psychological, and social capacities [2], [3]. The direct cost of falling exceeds $10 billion a year in the United States [1], [2], with almost 9,500 deaths per year attributed to falling [2], [3]. Epidemiological studies have shown that 30–70% of falls occur while level walking and thus understanding balance control during gait remains paramount.

Falls in the elderly are a complicated phenomenon comprising multifactoral risk factors, including both intrinsic and extrinsic issues [4]. Intrinsic factors, or those related to the individual, include a decreased performance in the balance control system, with loss of mobility being a strong indicator for increasing fall risk. In order to maintain stability, adequate levels of vision, vestibular function, musculoskeletal function, and proprioception are all required. Prior studies have also shown that decreased lower-extremity muscle strength and cognitive function are significant predictors of falls among older adults [4]–[6]. Extrinsic factors, or those pertaining to environmental hazards, contribute significantly to fall incidents and can include objects to trip over, poor lighting, slippery surfaces, or inappropriate furniture [3]. The ability to understand which of the multitude of neuromuscular, cognitive, and sensory factors most contribute to balance control ability during gait can provide further ability to diagnose and treat elderly at risk of falling.

While clinically valuable, gait analysis can be both expensive and time inefficient for laboratory technicians, with a data collection taking up to 2 hours and costing up to $2,000 [7]. Having a model that could predict the fall risk of elderly individuals based on calculated gait balance control parameters would be a clinically viable and inexpensive solution. In order to achieve this, models are needed which can find a mapping between clinical and laboratory biomechanical measures. Fall prediction models have previously used logistic regression as well as static posture variables and clinical measures to determine fall risk. These included predictions based on Berg balance scores, Timed Up and Go test, and self-reported history of imbalance and history of falls to determine the risk of falling among a group of elderly individuals [4], [8]. Such models require that input predictors explain a high degree of variability and make the assumption that linear relationships exist between variables. Another approach which would allow for non-linear relationships and include a number of input variables is an artificial neural network (ANN) [9]. An advantage of ANN models is that they can be built to infer a function simply from observation or training. By exposing the model to set of elderly adult data, with known input and output values, the ANN can be trained to an appropriate level.

Neural networks have been previously trained to efficiently determine foot-strike and foot-off events [10], as well as identify temporal or amplitude asymmetry in bilateral vertical ground reaction forces [11]. The use of an ANN in gait analysis has additionally demonstrated greater accuracy in discriminating patient populations from healthy adults, when compared to using linear discriminant analysis [12]. Further applications include estimating joint kinetics and kinematics using electromyography [13], as well as mapping spatio-temporal gait and electromyographic measures to dynamic balance control measures and fall risk [9], [14].

Since complete and accurate measurement of spatio-temporal gait variables is not possible in the clinical environment, the purpose of this study was to test the feasibility of a neural network model in mapping commonly used clinical measures to laboratory balance measures. Clinical measures included a history of falls, deficits in sensory motor function, visual and hearing impairment, presence of chronic disease or depression, number of medications, and clinical balance examinations. We hypothesized that an ANN model could determine the balance control of elderly individuals given easily assessable clinical measures such as static balance examinations, cognitive performance, and muscle strength.

## Methods

### Subjects

A total of 56 community living elderly subjects [age (SD)  = 76.1 (6.5) years; 22 males] were recruited for this study. A phone screen was performed prior to recruitment. All subjects reported no history of head trauma, neurological disease, heart disease or visual impairment that was uncorrected by glasses. In addition, subjects confirmed that they were able to ambulate for up to 10 minutes without the use of an assistive device. A clinical and laboratory gait evaluation was then performed on all subjects by a physician and trained researchers, respectively. Each subject signed an informed consent statement, in accordance with ethics approval granted from the Institutional Review Board of University of Oregon, prior to participation in the study.

### Clinical Evaluation

The body mass index (BMI) was computed for each subject along with a full medical history of prior fall history, the number of medications taken, and co-morbidities. In addition, physicians evaluated proprioceptive ability, vision, and hearing. The Geriatric Depression Scale (GDS) was used to evaluate depression [15]. The Activities Specific Balance Confidence Scale (ABC) provided information on a person's self-perception of balance ability [16]. Static balance was evaluated using the Berg Balance Scale (BBS) [17]. Dynamic gait performance was recorded through the Timed Up and Go test (TUG) [18]. Cognitive ability was estimated using the Trail Making Test (TMT) A and B, as well as the Saint Louis University Mental Status (SLUMS). The TMT test was evaluated based on the difference in scores on the B and A test [19]. This difference has been shown to demonstrate the task switching cost. The SLUMS was used to identify any dementia or mild neuro-cognitive disorder by conducting screening tests for orientation, memory, attention, and executive function [20].

Bilateral isometric muscle strength of the hip abductors, knee extensors, and ankle plantarflexors was tested using a Biodex System 3 dynamometer (Biodex Medical Systems, NY). For hip strength, the subject was instructed to abduct while standing in the neutral position. Knee extensor strength was evaluated in the seated position at 60 degrees of knee flexion. Ankle plantorflexor strength was tested while seated at 20 degrees of knee flexion and in a neutral ankle position. The peak torque value for each joint was recorded and normalized to a person's body mass.

### Laboratory Gait Balance Evaluation

Subjects were asked to walk at a self-selected comfortable speed across a 10-meter walkway. During ambulation, 29 retro reflective markers were placed on bony landmarks of the body [21], with three dimensional marker trajectories captured with an 8-camera motion analysis system (Motion Analysis Corp, Santa Rosa, CA). Data were filtered using a fourth-order low pass Butterworth filter with an 8-Hz cutoff frequency. Ground reaction forces and moments were captured from three floor-embedded force plates (Advanced Mechanical Technologies Inc., Watertown, MA). Marker and force plate data were collected at 60 Hz and 960 Hz, respectively.

Balance control during gait included analysis of the position and velocity of the center of mass (CoM) in relation to the dynamically changing base of support (BoS) [22]. The distance from the CoM position to the closest border of the BoS (CoM-BoS) represented static balance control. The displacement of the CoM along the direction of the CoM velocity vector to the boundary of the BoS (CoMv-BoS) represented dynamic balance control. The BoS area was calculated based on the anthropometrics and configuration of the feet. These three measures were evaluated at heel strike of both limbs across all gait cycles.

### ANN Development

An artificial neural network is a series of interconnected nodes (biological neurons) which approximates the relationships, or adaptive weightings, between input and output measures. Similar to biological nervous systems, connections (biological synapses) were established through a learned iterative process. Upon receiving one or more inputs (biological dendrites), a node was able to compute a weighted sum and pass a value through a non-linear transfer function to establish an output function. Training, or learning and the establishment of synapses, occurred by using the clinical (inputs) and balance control (outputs) data among a subset of individuals, then solving for the weights of the inter-connections in an optimal manner. Inferring the mapping implied by the data and finding the solution that has the smallest possible cost allows the ANN to arrive at a satisfactory weighting level. Once the ANN model was trained, it was then be used to predict outputs for the remaining subset of individuals.

The ANN used in this study was designed to calculate the gait balance control measures of each subject. Input data sets included subject characteristics (age, BMI, gender), clinical examination (fall history, medications, vision, hearing), clinical balance performance (BBS, TUG, ABC), cognitive evaluation (TMT, GDS, SLUMS), and muscle strength (bilateral ankles, knees and hips). The ANN program is provided as Program S1 in supplementary materials.

**Figure 1:**
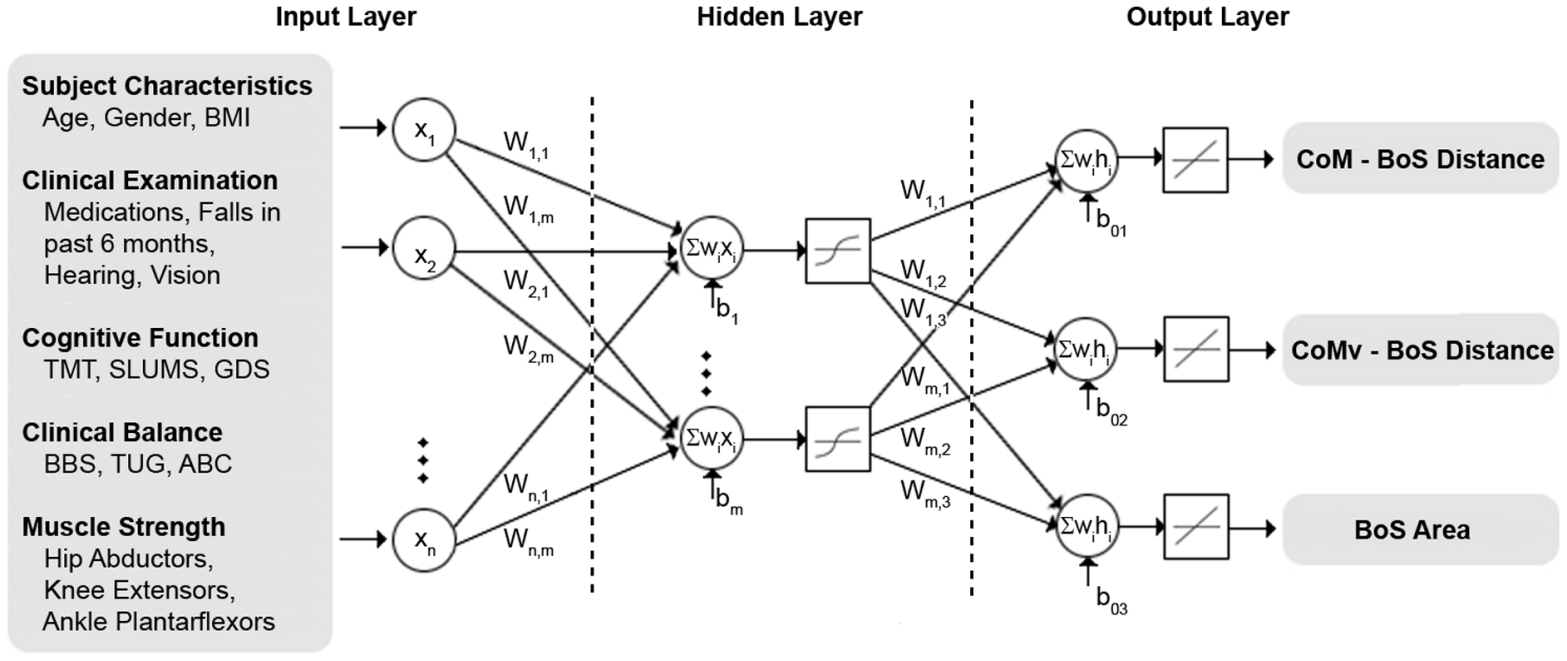
Neural network architecture representing the three layers as well as the tangential sigmoid and pure linear transfer functions in the hidden and output layers, respectively. All nodes are not represented in this diagram, though a weighted sum of all inputs and the bias is performed at each node in the hidden and output layers.

A three-layer, feed-forward back-propagation ANN was constructed using MATLAB (Mathworks Inc., Natick, MA; Figure 1 Program S1). The first layer of the network consisted of different combinations of the normalized input data sets, with between three and 16 possible clinical measures included in each iteration of the analyses. The second layer included 5, 10, 20 or 30 hidden neurons. The third or output layer included the three laboratory gait balance control variables. Out of the 56 subjects, 42 were randomly selected for training, with testing performed on the other 14 subjects. This process was repeated 4 times in order to test the network on all 56 subjects, with training stopped when the mean squared error (MSE) error reached 0.1, 0.01 or 0.001. Error correction during training was conducted with the Levenberg-Marquardt algorithm [23]. Weighted incoming signals were summed at the hidden and output units, with a tangential sigmoid transfer function and pure linear transfer function used at each layer, respectively. Details of the network have been described previously by Hahn and colleagues [14].

After successful training, all balance control data was converted back to real world units of cm and cm^2^, for the distance and area measures, respectively. The ability of the ANN model to accurately estimate CoM-BoS balance control measures in comparison to actual gait measurements was assessed via correlation analysis. Differences in accuracy in the correlation coefficient (R) between the number of hidden units (5, 10, 20 or 30), between the error goal (0.1, 0.01 and 0.001), and across grouping type were assessed with a 3 way ANOVA in SPSS 14.0 (IBM Inc., Armonk, NY).

## Results

**Figure 2:**
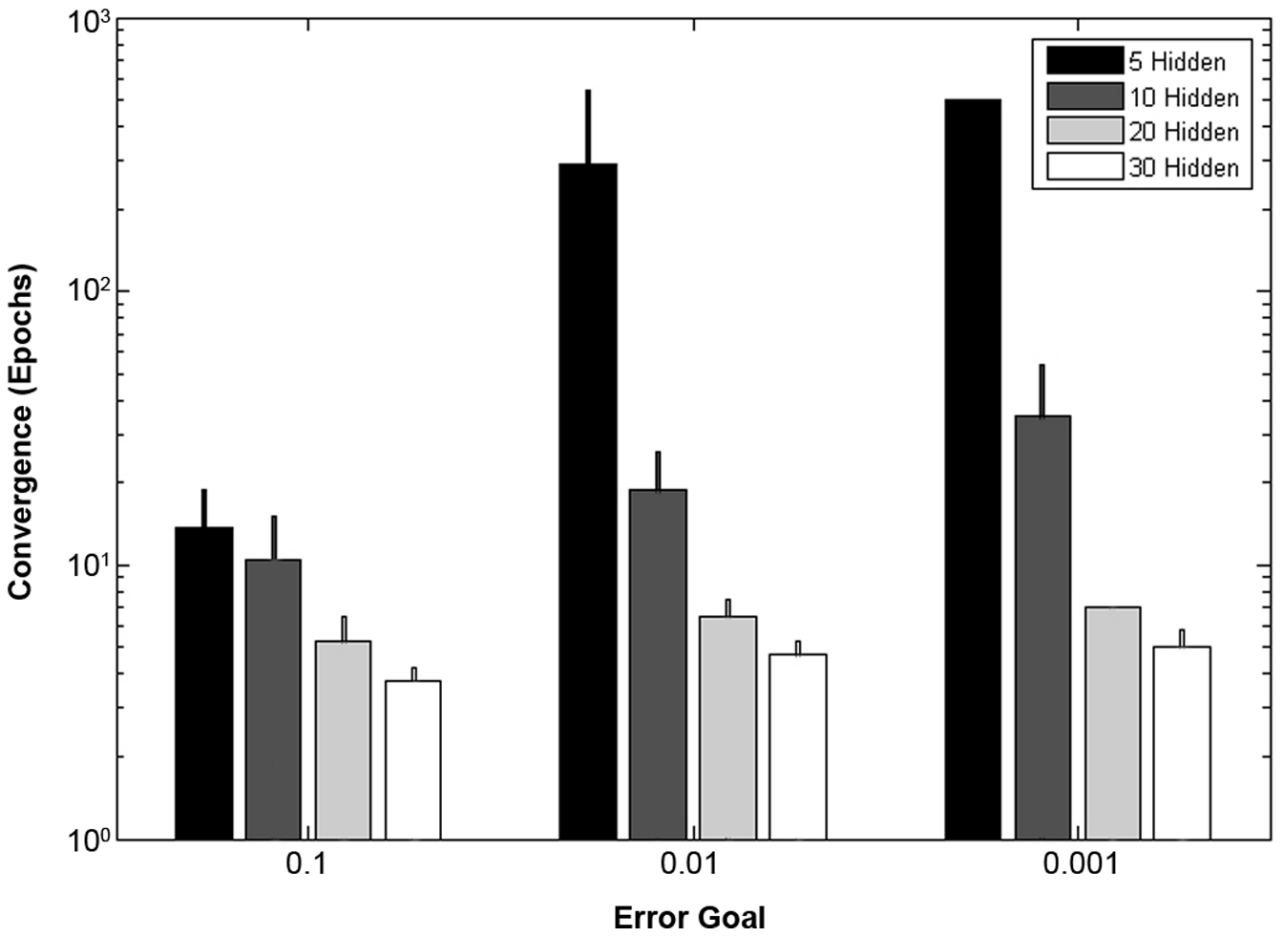
Number of epochs required for convergence to error goal given the number of hidden nodes during training of the neural network with all 16 input variables.

The ability to calculate gait balance control using five functional domains as well as a combination of all variables were investigated (Table 1). In addition, 4 different hidden node sizes and 3 MSE error goals were assessed for a total of 72 network iterations. Minimal processing time was required for network training on all these combinations. When 5 hidden nodes were used, much greater time was needed for the solution to converge to an MSE error of less than 0.01 or 0.001, with much of the samples reaching the maximum limit of 500 epochs before failing to reach the goal (Figure 2). The use of 20 or 30 hidden nodes was much more efficient in training the data sets at all error goals.

**Table 1:** Demographics of all 56 participants [mean (SD)].

Subject Characteristics		
	Age (years)	76.1 (6.5)
	Gender (Males/Females)	22/34
	BMI	27.4 (6.1)
Clinical Examination		
	Fall History (number in past year)	0.95 (1.35)
	Number of Medications	3.8 (3.2)
	Visual acuity (/20)	36.6 (11.7)
	Hearing (number impaired)	14
Clinical Balance		
	BBS (/56)	53.4 (3.8)
	TUG (seconds)	9.0 (2.0)
	ABC (%)	85.7 (13.6)
Cognitive Performance		
	TMT B-A (seconds)	63.2 (63.0)
	GDS (/15)	1.6 (1.9)
	SLUMS (/30)	26.4 (3.3)
Muscle Strength a		
	Ankle Plantarflexion	3.1 (2.3)
	Knee Extension	3.8 (2.7)
	Hip Abduction	1.9 (1.6)
Gait Balance Control b		
	CoM-BoS distance (cm)	3.8 (1.1)
	CoMv-BoS displacement (cm)	19.3 (3.5)
	BoS Area (cm^2^)	436 (88)

a Normalized to body weight and body height (Nm/BW*BH).

b Balance control measures evaluated at heel strike.

**Figure 3:**
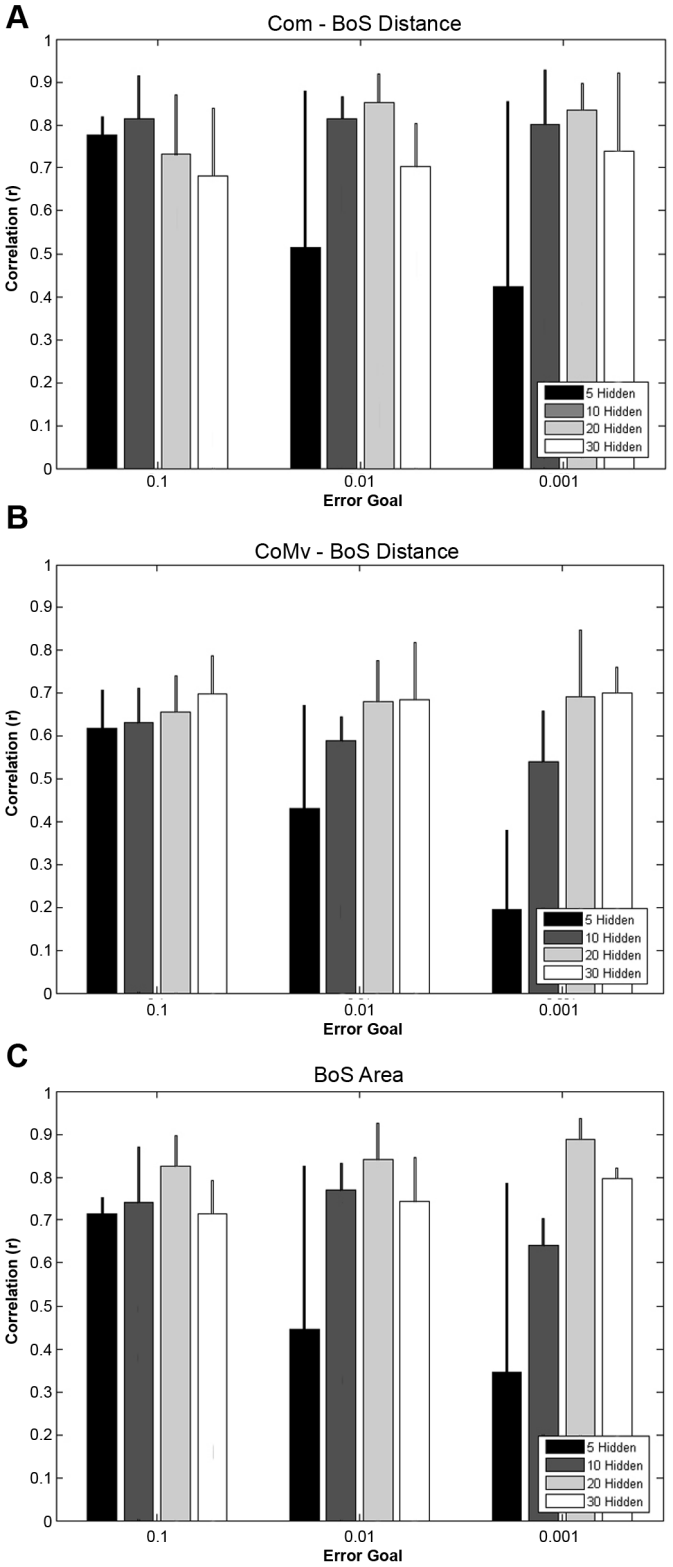
Performance of the neural network using all 16 input variables.

The input type by error goal by hidden nodes interaction was not detected for the CoM-BoS (P = .849), CoMv-BoS (P = .877) or BoS Area (P = .477) correlations. Alternatively, an error goal main effect was demonstrated for all three balance control dependent variables (P<.001). Overall, as the error goal was decreased from 0.001 to 0.1 there was an increase in the correlation coefficient (Figure 3), especially when utilizing 5 hidden nodes. Additionally, an increase in hidden nodes from 10 to 20 demonstrated on average a 0.10 greater correlation for the BoS area (P = 0.008) and a 0.08 greater correlation for the CoM-BoS distance (P = 0.057). Increasing to 30 hidden nodes demonstrated 0.08 and 0.11 greater correlation for the CoMv-BoS distance and BoS area, respectively (P = 0.016 and P = 0.004). No other hidden node or error goal differences were detected.

**Figure 4:**
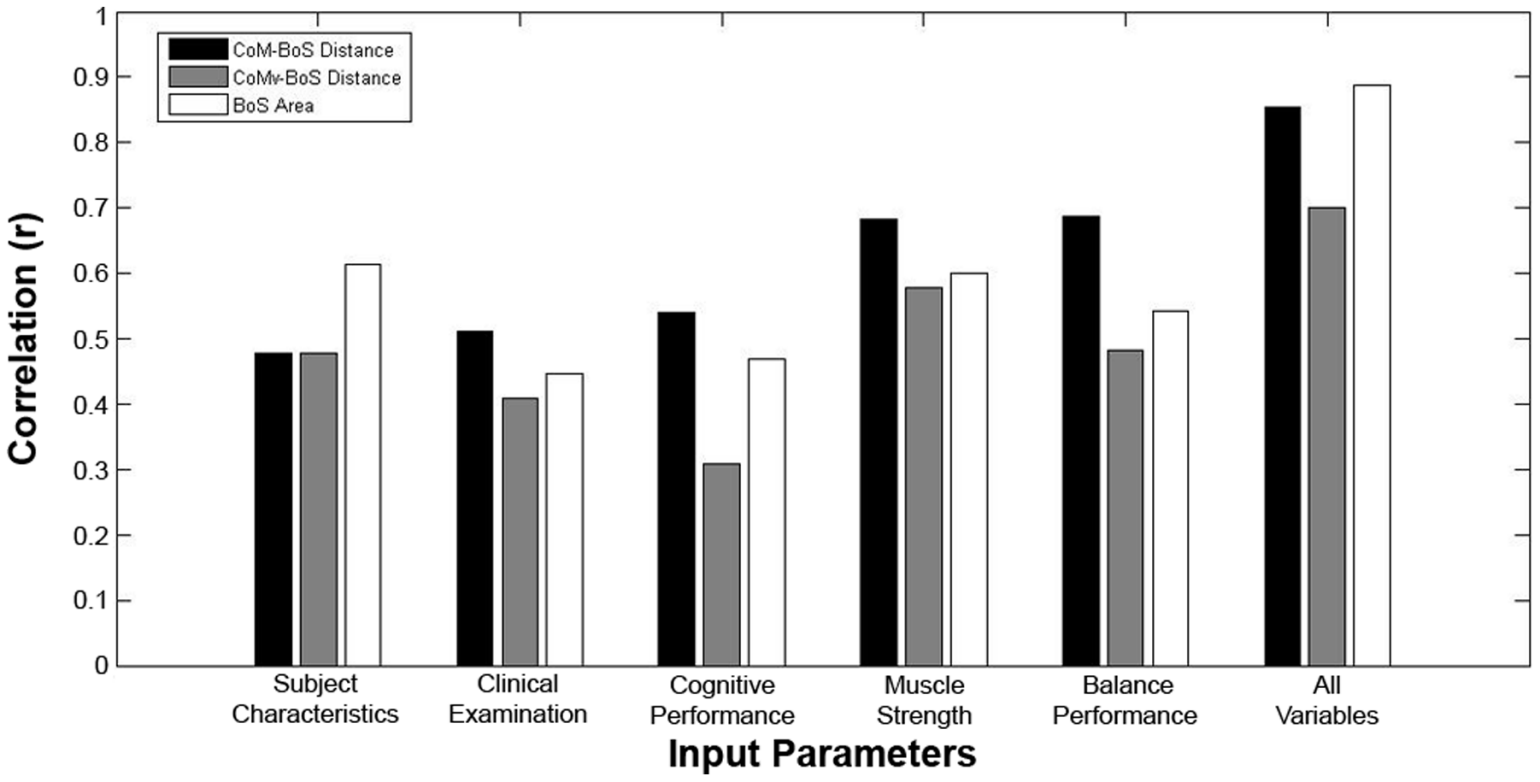
Maximum mapping performance of a three layer neural network in estimating the CoM-BoS distance, CoMv-Bos displacement and BoS area across the five different input variable categories as well as when using a combination of all input categories.

**Figure 5:**
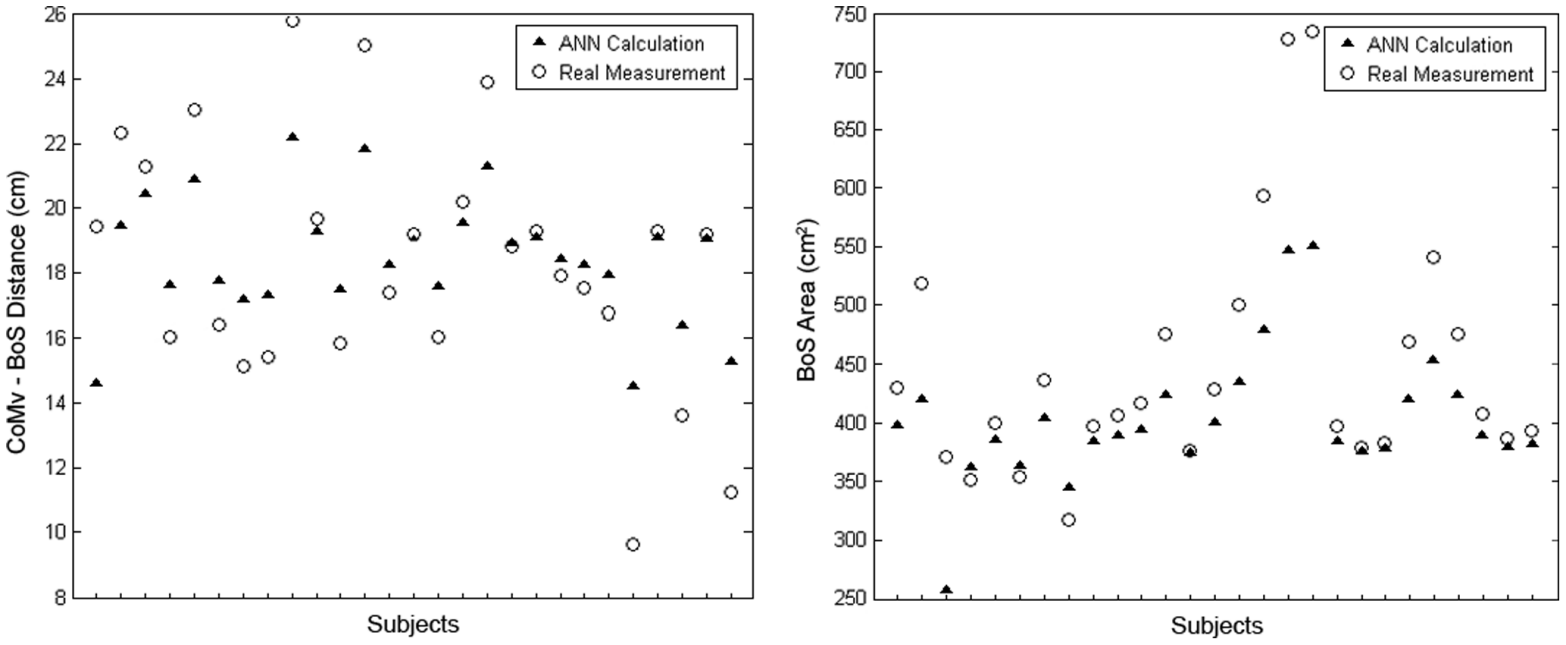
Representative data for the CoMv-BoS distance (A) and the BoS Area (B), as calculated by a neural network (triangles) with 20 hidden nodes and an error goal of 0.01. All input variables were included in this training set, with the actual values for these balance control measures represented by the open circles.

Input variable differences were also demonstrated, as greater correlations were demonstrated by using all variables, when compared to any single input type (Figure 4). The combination of all input variables, with 20 hidden nodes and a 0.001 error goal resulted in the best training across all three dependent variables (Table 2). The use of these parameters provided convergence within an average of 4 epochs to finish training the network and provided correlation values of R>0.80 for the CoM-BoS distance and BoS Area. On average, using all input variables, the ANN was able to calculate the CoMv-BoS distance to within 1 cm and the BoS Area to within 75 cm^2^ for elderly adults (Figure 5).

**Table 2:** Average performance (SD) of selected combinations of inputs and the corresponding hidden nodes and error goal values that produced the highest accuracy.

Inputs	Hidden Nodes	Error goal	R^1^	R^2^	R^3^
All Input Variables	20	0.001	0.84 (0.06)	0.69 (0.16)	0.89 (0.05)
Clinical Balance and Clinical Exams	20	0.001	0.72 (0.22)	0.63 (0.12)	0.72 (0.10)
Clinical Balance and Cognitive Tests	20	0.01	0.67 (0.11)	0.54 (0.13)	0.63 (0.17)
Clinical Balance and Muscle Strength	20	0.01	0.74 (0.08)	0.73 (0.05)	0.63 (0.10)
Clinical Exams and Muscle Strength	20	0.01	0.71 (0.14)	0.57 (0.18)	0.72 (0.07)
Cognitive Tests and Muscle Strength	30	0.1	0.56 (0.08)	0.68 (0.04)	0.51 (0.05)

1 Correlations for the CoM-BoS distance.

2 Correlations for the CoMv-BoS displacement.

3 Correlations for the BoS Area.

Utilizing all input variables, variability in input weights were demonstrated across all learning iterations of the neural network. While the predictive nature of the input weights is unknown, nonetheless, the largest weights in the input layer were found for the ABC test, vision performance, and hip abductor strength.

## Discussion

The purpose of this study was to demonstrate that given clinical measures readily obtained by a physician, an artificial neural network can determine gait balance control among elderly adults ambulating in a laboratory. In support of our hypothesis, with the use of subject characteristics, clinical examinations, cognitive evaluations, and muscle strength, we were able to demonstrate that an ANN model could determine the balance control of elderly individuals during gait.

Utilizing a combination of all variables performed strongest in this study with correlation values for mapping clinical to balance control measures of up to 0.89. Among the various functional domains, muscle strength and clinical balance measures correlated to gait stability better than subject characteristics, clinical examinations, and cognitive performance. As the musculoskeletal system is the effector system which maintains posture and controls movement [24], it understandably plays an important role in predicting dynamic balance control. Among nursing home residents with a history of falls, the peak torque and power of knee extensors, knee flexors, ankle plantarflexors, and ankle dorsiflexors were significantly less than those of age-matched controls [25]. Similarly, clinical balance measures such as the BBS, though a measure of static balance ability, also maps strongly to dynamic CoM and BoS interactions during gait. Additionally, both muscle strength tests and clinical balance examinations are commonly used in the clinical setting to evaluate elderly adults at risk for falling.

As individual functional domains, subject characteristics, clinical examinations and cognitive performance did not map strongly to gait balance control performance. While age related differences have been reported for the BBS, TUG, and gait speed for male and female older adults [26], these measures were not strongly correlated with gait balance control using the ANN mapping, as R values for these three domains ranged from 0.3 to 0.6. Interestingly, a combination of the TMT, SLUMS and GDS did not demonstrate strong mapping with gait balance control measures as well. Though depression has been associated with standing imbalance [27] and increased incidence of falls [28], similar relationships to gait balance control were weak. Similarly, medication use, prior falls and cognitive performance have all shown a relationship to falls, though they were not as strong when mapping to dynamic balance control [28]. Among our subject population though, most adults reported no depression, while being highly active and functional. Confirming the findings of the two strongly correlated functional domains, Rubenstein found that the important risk factors for falls are more often related to muscle weakness and gait or balance deficits [29].

Improvement in the ability to properly determine balance control measures were demonstrated with an increased number of hidden units. The use of additional hidden nodes has previously been hypothesized to be an indicator of enhanced generality, with greater plasticity and pathways to a solution [14]. Similar network architecture has been successful in gait research. The ability to characterize lower extremity joint kinematics and kinetics based on muscle electromyographic activity was shown to confirm with physiological expectations [13]. Prior studies have also utilized two hidden layer architectures and shown an ability to correctly identify gait conditions using fast Fourier transform of lower extremity kinematics as inputs, with up to 83% accuracy [30]. In the current study, single hidden layer architecture was utilized as this has been shown to be computationally faster and sufficient for learning functional relationships [31].

While neural network weights are variable and the predictive strengths unknown, the ABC score, vision, and hip abductor strength demonstrated the greatest weighting when all groupings were included as network inputs. The ABC, which is sometimes used as an indicator of fear of falling, has also been shown previously to be sensitive in discriminating fallers from non-fallers [32]. Similarly, the ability to maintain balance is a function of adequate visual information, with Nashner and Berthoz (1978) demonstrating that reduction in vision increased sway amplitude among older adults [33]. Furthermore, the hip abductor has been shown to be important in maintaining lateral stability, with changes in the base of support adapted by older adults in order to control the CoM and compensate for decreased hip abductor strength [34].

The use of biomechanics laboratory equipment to assess gait performance can be time consuming and expensive [7]. While biomechanical data are essential for determining the underlying mechanisms of balance impairment and possible fall incidents [35], the ability to categorize and identify community dwelling elderly fallers at risk of falls in a timely and inexpensive manner is needed. The strength of this study is the ability to map clinical measures routinely collected by physicians to dynamic gait measures which better characterize a person's balance control. The advantages of using an ANN is the ability to reveal the multifactorial factors that can lead to poor balance control during gait. By including a combination of five functional domains, it is possible to learn mappings from the clinical measures to dynamic balance control, and apply these connections to novel data sets.

A limitation of this study included the small sample size. Though only 56 adults have thus far been fully screened by a physician, the use of a neural network still demonstrated the ability to quickly be trained and showed high correlation values of up to 0.89. This provides further evidence that an ANN can successfully be used to assess a person's gait balance control, without the need for full assessment within a laboratory setting. Future research needs to investigate the generalizability of this algorithm to a larger sample of older adults. Additionally, utilization of a neural network to predict changes in balance control ability and fall risk in the elderly based on different interventions would be beneficial. The ability for the network to provide predicted balance control outcomes based on improvements at the input layer, such as alterations in muscle strength, medications, or cognitive ability, will hopefully provide a quick and useful way to assess predicted changes in gait performance and possible fall risk. Similar assessments of fall risk are available for clinical examinations such as the BBS [4] or TUG [8], but a generalized form including all five domains would be a valuable tool for older adults and physicians.

In conclusion, results from this study demonstrated that an artificial neural network could be trained to map clinical variables to biomechanical measures of gait balance control. While further studies will investigate the generalizability of this network to a larger group of subjects, these initial findings suggest that an ANN can be used to assess balance impairment in the elderly.

## Supporting Information

Program S1 **MATLAB codes for the three-layer, feed-forward back-propagation ANN.**
(DOCX)
