# Supplementary material for: An Artificial Neural Network Estimation of Gait Balance Control in the Elderly Using Clinical Evaluations
Source: PLoS One. 2014 May 16;9(5):e97595. doi: 10.1371/journal.pone.0097595 (PMC4023967; doi:10.1371/journal.pone.0097595)
Supplement: Program S1 — MATLAB codes for the three-layer, feed-forward back-propagation ANN. (DOCX) [file pone.0097595.s001.docx]

% NN_clin_bos.m

%

% Vipul Lugade and Li-Shan Chou, Ph.D.

% 5/10/11

% Motion Analysis Laboratory

% Department of Human Physiology

% University of Oregon

%

% Purpose: To test clinical inputs and predict biomechanical outputs

%

% Utilizes: Neural network Matlab toolbox

%

% requires input and output data to work:

% for input (in_file) utilizing 'Clinical-Input.dat', which will have 16

% unlabeled columns of data for all functional domains (age gender bmi bbs

% falls tug tmt abc meds vision hearing gds slums ankleStrength

% kneeStrength hipStrength)

% for output (out_file) utilizing 'BoS-Output.dat', which includes base of

% support versus center of mass variables (com-bos, comv-bos, bos_area)

%

% Lines to modify:

% Uncomment the required line for the variable "net_input" to test the

% neural network under different domains (subject characteristics, clinical

% tests, clinical balance, cognitive, strength or combinations of these)

% Lines 44-58 if you are using certain functional domains

%

clear;

close;

tic

%% Initialize variables

% input for into neural net

subjpath = 'C:\Users\vips\Documents\Motion Lab\Dissertation\ANN\Clinical - Biomech\All Subjects\';

in_file = 'Clinical-Input.dat';

net_input = load([subjpath,in_file]);

out_file = 'BoS-Output.dat';

desired_out = load([subjpath,out_file]);

% selecting com-bos, comv-bos and bos_area

desired_out = desired_out(:,[1 2 3]);

% Variables to select from

% age gender bmi bbs falls tug tmt abc meds vision hearing gds slums

% lank/rank lknee/lknee lhip/rhip

% functional domains:

% subject characteristics = [age gender bmi]

% net_input = net_input(:,[1 2 3]);

% clinical/sensory = [falls meds vision hearing]

% net_input = net_input(:,[5 9 10 11]);

% balance = [BBS TUG ABC]

% net_input = net_input(:,[4 6 8]);

% cognitive = [TMT GDS SLUMS]

% net_input = net_input(:,[7 12 13]);

% muscle strength = [ankle knee hip]

% net_input = net_input(:,14:16);

% strength + clin

% net_input = net_input(:,[14 15 16 5 9 10 11]);

% all

net_input = net_input(:,1:16);

% determine number of trials and inputs

numTrial = length(net_input(:,1));

numIn = length(net_input(1,:));

% number of output variables

numOut = length(desired_out(1,:));

if length(desired_out(:,1)) ~= numTrial

error('Number of Inputs and Outputs do not match');

end

% number of hidden nodes

numHid = [5 10 20 30];

% mse error to train until

error_goal = [0.1 0.01 0.001];

% Randomize all of the subjects

rand_subj = randperm(numTrial);

% Performing 9 iterations on the data since will be training 24/testing 3

% unique each time. if 3 iterations train 18/test 9

% = 4, when have 56 subjects and doing 75/25% train/test

numLoop = 4;

% use mapstd to convert data to z scores

[in, in_process] = mapstd(net_input');

% output also zscored since using purelin as the output transfer function.

[out, out_process] = mapstd(desired_out');

%{

% if we want to scale between 0 and 1 or -1 and 1 will use tansig or logsig

% as the output transfer function

% discretize the output data to be between -1 and 1

out_min = min(desired_out);

out_max = max(desired_out);

out = zeros(numTrial,numOut);

for i = 1:numOut

out(:,i) = 2*(desired_out(:,i)-out_min(i))/(out_max(i)-out_min(i))-1;

end

out = out';

%}

% initialize all the important outputs 3D to take into account the loops,

% the numHid and numErrorgoals

save_train_perf = cell(3,3);

save_train_result = cell(3,3);

save_test_perf = cell(3,3);

save_test_result = cell(3,3);

save_real_out = cell(3,3);

save_subj = cell(3,3);

save_R = cell(3,3);

save_P = cell(3,3);

save_mse = cell(3,3);

% save the net object into a structure

count = 1;

% net_struct = struct([]);

for H = 1:length(numHid) %test all 3 hidden unit sizes

for E = 1:length(error_goal) % test all three error goals

% save each loop through individually

train_perf = cell(numLoop,1);

train_result = cell(numLoop,1);

test_perf = zeros(numLoop,1);

test_result = cell(numLoop,1);

real_out = cell(numLoop,1);

% R and P have 3 output variables (for correlations)

R = zeros(numLoop,3);

P = zeros(numLoop,3);

mse_real = cell(numLoop,1);

for i = 1:numLoop

%% SETUP Network

% select the subjects to test and train with for this iteration

cols = rand_subj;

% determine the number of subjects to test with.

numTest = numTrial/numLoop;

% select the testing subjects

test_cols = cols((i-1)*numTest+1:i*numTest);

test_in = in(:,test_cols);

test_out = out(:,test_cols);

train_in = in;

train_out = out;

train_in(:,test_cols)=[];

train_out(:,test_cols)=[];

% use newff function to setup the network

net = newff(train_in,train_out,numHid(H),...

{'tansig' 'purelin'},'trainlm');

% trainlm = Levenberg-Marquardt algorithm

% tansig = tangential sigmoid function

% purelin = pure linear transfer function at output level

%

%{

net = newff(P,T,S,TF,BTF,BLF,PF,IPF,OPF,DDF)

NEWFF(P,T,S,TF,BTF,BLF,PF,IPF,OPF,DDF) takes,

P - RxQ1 matrix of Q1 representative R-element input vectors.

T - SNxQ2 matrix of Q2 representative SN-element target vectors.

Si - Sizes of N-1 hidden layers, S1 to S(N-1), default = [].

(Output layer size SN is determined from T.)

TFi - Transfer function of ith layer. Default is 'tansig' for

hidden layers, and 'purelin' for output layer.

BTF - Backprop network training function, default = 'trainlm'.

BLF - Backprop weight/bias learning function, default = 'learngdm'.

PF - Performance function, default = 'mse'.

IPF - Row cell array of input processing functions.

Default is {'fixunknowns','remconstantrows','mapminmax'}.

OPF - Row cell array of output processing functions.

Default is {'remconstantrows','mapminmax'}.

DDF - Data division function, default = 'dividerand';

and returns an N layer feed-forward backprop network.

The transfer functions TF{i} can be any differentiable transfer

function such as TANSIG, LOGSIG, or PURELIN.

The training function BTF can be any of the backprop training

functions such as TRAINLM, TRAINBFG, TRAINRP, TRAINGD, etc.

%}

net.trainParam.goal = error_goal(E);

net.trainParam.epochs = 500;

% change this to display only at the end... can reduce if you

% want real time updates

net.trainParam.show = 500;

% training 90% of the in subjects randomly chosen by me above,

% will test the other 10% using sim later.

net.divideParam.trainRatio = 1;

net.divideParam.valRatio = 0;

net.divideParam.testRatio = 0;

%% train the network

[net, tr, Y] = train(net,train_in,train_out);

train_perf{i} = tr.perf;

train_result{i} = Y;

% save the net

net_struct(count) = struct(net); %#ok<SAGROW>

count = count+1;

%{

[net,tr,Y,E,Pf,Af] = train(NET,P,T,Pi,Ai)

TRAIN trains a network NET according to NET.trainFcn and

NET.trainParam.

TRAIN(NET,P,T,Pi,Ai) takes,

NET - Network.

P - Network inputs.

T - Network targets, default = zeros.

Pi - Initial input delay conditions, default = zeros.

Ai - Initial layer delay conditions, default = zeros.

and returns,

NET - New network.

TR - Training record (epoch and perf).

Y - Network outputs.

E - Network errors.

Pf - Final input delay conditions.

Af - Final layer delay conditions.

%}

%% test the network with 10%

[test_result{i},Pf,Af,test_E,test_perf(i)] = sim(net,...

test_in,[],[],test_out);

%{

SIM simulates neural networks.

[Y,Pf,Af,E,perf] = SIM(net,P,Pi,Ai,T) takes,

NET - Network.

P - Network inputs.

Pi - Initial input delay conditions, default = zeros.

Ai - Initial layer delay conditions, default = zeros.

T - Network targets, default = zeros.

and returns:

Y - Network outputs.

Pf - Final input delay conditions.

Af - Final layer delay conditions.

E - Network errors.

perf - Network performance.

%}

%% Convert back to real world numbers

% if using 0 mean unity sd, use the mapstd function

real_out{i} = mapstd('reverse',test_result{i},out_process);

% if discretized use the following

% real_out{i} = ((test_result{i}+1)/2)*

% (out_max(i)-out_min(i))+out_min(i);

% calculate correlation of output vs actual output

all_NN = [real_out{i} mapstd('reverse',Y,out_process)];

all = [mapstd('reverse',test_out,out_process) ...

mapstd('reverse',train_out,out_process)];

[temp_R, temp_P] = corrcoef(all_NN(1,:),all(1,:));

R(i,1) = temp_R(1,2);

P(i,1) = temp_P(1,2);

[temp_R, temp_P] = corrcoef(all_NN(2,:),all(2,:));

R(i,2) = temp_R(1,2);

P(i,2) = temp_P(1,2);

[temp_R, temp_P] = corrcoef(all_NN(3,:),all(3,:));

R(i,3) = temp_R(1,2);

P(i,3) = temp_P(1,2);

%mse of the outputs

mse_real{i}= mean((real_out{i}-mapstd('reverse',...

test_out,out_process)).^2,2);

end

%% save

save_train_perf{E,H} = train_perf;

save_train_result{E,H}= train_result;

save_test_perf{E,H} = test_perf;

save_test_result{E,H} = test_result;

save_real_out{E,H} = real_out;

save_subj{E,H} = rand_subj;

save_R{E,H} = R;

save_P{E,H} = P;

save_mse{E,H} = mse_real;

end % Error goal loop

end % Hidden nodes loop

toc
